# Non-Coding RNAs in Oral Cancer: Emerging Roles and Clinical Applications

**DOI:** 10.3390/cancers15153752

**Published:** 2023-07-25

**Authors:** Saurabh Dey, Bini Biswas, Angela Manoj Appadan, Jaladhi Shah, Jayanta K. Pal, Soumya Basu, Subhayan Sur

**Affiliations:** Cancer and Translational Research Centre, Dr. D. Y. Patil Biotechnology and Bioinformatics Institute, Dr. D. Y. Patil Vidyapeeth (DPU), Pimpri 411033, India; deysaurabh20@gmail.com (S.D.);

**Keywords:** oral cancer, head and neck cancer, non-coding RNA, cancer diagnosis, cancer therapy, microRNA (miRNA), long non-coding RNA (lncRNA), circular RNA (circRNA), PIWI-interacting RNA (piRNA), small nucleolar RNA (snoRNA)

## Abstract

**Simple Summary:**

Oral cancer (OC) is one of the most prevalent cancers in the world. Despite improvements in therapies, OC still has a poor survival rate of about 50%, with metastasis being the worst-case scenario. Thus, there is an urgent need to understand the disease process and to develop diagnostic and therapeutic strategies for OC. Advancement of high throughput genome sequencing shows that more than 90% of the human genome encodes non-coding transcripts that do not code for any protein. In this review, we discuss the role of various types of these non-coding RNAs (ncRNAs) in OC and their promising clinical implications. Dysregulated expressions of ncRNAs are associated with OC initiation and progression, as well as therapy resistance. Differential expressions of these ncRNAs in blood or saliva have indicated their potential diagnostic and prognostic importance. In this review, we have summarized all the promising aspects of ncRNAs in the management of OC.

**Abstract:**

Oral cancer (OC) is among the most prevalent cancers in the world. Certain geographical areas are disproportionately affected by OC cases due to the regional differences in dietary habits, tobacco and alcohol consumption. However, conventional therapeutic methods do not yield satisfying treatment outcomes. Thus, there is an urgent need to understand the disease process and to develop diagnostic and therapeutic strategies for OC. In this review, we discuss the role of various types of ncRNAs in OC, and their promising clinical implications as prognostic or diagnostic markers and therapeutic targets. MicroRNA (miRNA), long ncRNA (lncRNA), circular RNA (circRNA), PIWI-interacting RNA (piRNA), and small nucleolar RNA (snoRNA) are the major ncRNA types whose involvement in OC are emerging. Dysregulated expression of ncRNAs, particularly miRNAs, lncRNAs, and circRNAs, are linked with the initiation, progression, as well as therapy resistance of OC via modulation in a series of cellular pathways through epigenetic, transcriptional, post-transcriptional, and translational modifications. Differential expressions of miRNAs and lncRNAs in blood, saliva or extracellular vesicles have indicated potential diagnostic and prognostic importance. In this review, we have summarized all the promising aspects of ncRNAs in the management of OC.

## 1. Introduction

Oral squamous cell carcinoma (OSCC) or oral cancer (OC) is the most prevalent type of head and neck cancer that arises in the tongue, lips, and floor of the mouth. In the year 2020, GLOBOCAN estimated around 377,713 total cases and 177,757 deaths worldwide from lip and oral cavity cancer, in which India alone showed a high burden of the disease [1]. The American Cancer Society has recently estimated the incidence of cancers in the oral cavity and pharynx, with around 54,000 new cases and about 11,580 deaths in the year 2023 alone [2]. Some of the most common factors with which OC progression is often linked include high tobacco and alcohol consumption, as well as an infection caused by human papillomavirus (HPV) [3]. Despite the advancement of conventional therapeutic strategies, the overall survival rate of OC is barely 50%, and even worse in the case of metastasis [4]. Since 1991, the USA has shown a continuous decline in overall mortality by 33%. However, the mortality rate of OC has shown a continued increase by 2% in men and 1% in women per year [2]. The aggressiveness and heterogeneity of the disease with delayed diagnosis, lack of early detection markers, lack of effective chemotherapeutic drugs, therapy resistance, and side effects often make the management of the disease complicated [5]. Although the U.S. Food and Drug Administration (FDA) approved EGFR targeted therapy and PD-1/PD-L1 immune therapy show some promising results, but these have achieved limited success [6,7]. Thus, there is an urgent need to understand the disease process and to develop better diagnostic and therapeutic strategies.

**Figure 1:**
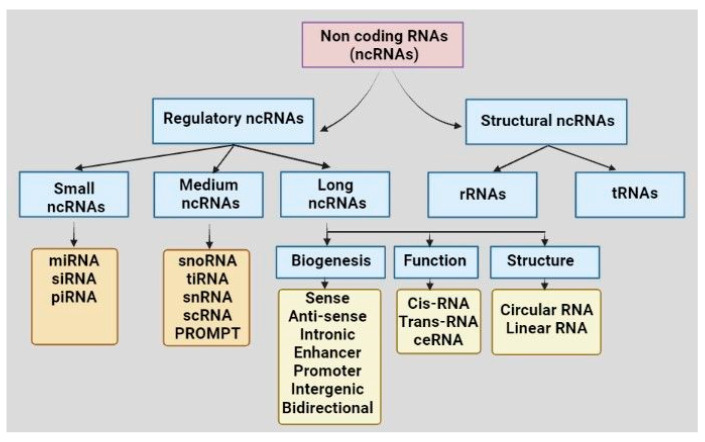
Classification of non-coding RNAs based on structures and functions. Abbreviations: non-coding RNA: ncRNA, rRNA: ribosomal RNA, tRNA: transfer RNA, miRNA: microRNA, piRNA: PIWI-interacting RNA, siRNA: small interfering RNA, snoRNA: small nucleolar RNA, tiRNA: transcription initiation RNA, snRNA: small nuclear RNA, scRNA: small cytoplasmic RNA, ceRNA: competing endogenous RNA, PROMPT: promoter upstream transcript.

Recent developments in high throughput genome sequencing have revealed that more than 90% of the human genome encodes non-coding transcripts that do not code for any protein [8]. For a long while, non-coding RNAs (ncRNAs) were considered junk materials for cells. Recent knowledge on comprehensive molecular evaluations has led to the noncoding genes acquiring considerable attention nowadays in the advancement of several diseases, including cancers. Based on structural and functional characteristics, types of ncRNAs present in a cell are tRNA, rRNA, long non-coding RNA (lncRNA), circular RNA (circRNA), microRNA (miRNA), PIWI-interacting RNA (piRNA), and small nucleolar RNA (snoRNA) (Figure 1). The ncRNAs are sometimes tissue-specific and cellular-compartment-specific in their distribution and expression, and sometimes ubiquitous. NcRNAs are found to interact with nucleic acids or proteins to alter their conformation, activities, and stabilities. The differential expressions of ncRNAs are reported in different cancers. For example, miR-15/16, miR-29, miR-34, miR-200 family, let-7, miR-21, miR-155, miR-17–92 cluster, miR-221/222, miR-195 and miR-26b were significantly modulated in different cancers, including leukemia, prostate cancer, colorectal cancer, pancreatic cancer, liver cancer, lung cancer, breast cancer, glioblastoma, ovarian cancer, renal cancer, and thyroid cancer, and involved in cancer progression, metastasis, and drug resistance [9,10]. The high expression of miR-195 and miR-26b and down-regulation of their common target gene Semaphorin 6D (SEMA6D) were found to be associated with therapy resistance in breast cancer and, thus, this signaling axis is suggested as a predictive marker for chemotherapy response [10]. Likewise, up-regulation of lncRNA HOTAIR and MALAT1, down-regulation of lncRNA Meg3 and dual function of lnRNA H19 were seen in different cancer types, including lung cancer, ovarian cancer, prostate cancer, breast cancer, colorectal cancer, gastric cancer, and liver cancer [9]. Among the circRNAs, circPRKCI and circHIPK3 were found to be up-regulated in glioma, lung cancer, breast cancer, colorectal cancer, gallbladder cancer, gastric cancer, and ovarian cancer [9]. Differential expressions of piR-651, piR-823, piR-932 were reported in cancers of lung, breast, colorectal, esophageal, and gastric cancer [9]. Apart from the primary tissues, differential expressions of the ncRNAs in body fluid, including blood and saliva, suggest their importance as diagnostic and therapeutic biomarkers.

To date, RNAseq studies have revealed more than 200 ncRNAs (including miRNA, lncRNA, circRNA, snoRNA, and piRNA) to have an association with OC progression. In this review, we have described the types of ncRNAs, their functions, and their possible role as diagnostic and prognostic markers in OC. The correlations between the expressions of these ncRNAs and OC have been elaborated further in this review. Results from different studies obtained so far suggest a better understanding of the relationship between non-coding RNA transcriptomic alterations and disease development, since these modifications have the prospective to be used as diagnostic and therapeutic biomarkers. Thus, the review has practical implications for the treatment of OC and may offer fresh perspectives and ideas for future mechanistic research in various preclinical and clinical settings.

## 2. Oral Cancer: Current Diagnostic and Prognostic Markers

Tissue biopsy and histological evaluation are the gold standard for diagnosing oral cancer (OC). However, this technique is painful for patients and causes a delayed diagnosis [11]. As non-invasive methods, metachromasia using iodine staining, or toluidine blue, which stains cancerous lesions, chemiluminescence-based lumenoscopy, auto-fluorescence based techniques like laser-induced auto-fluorescence (LIAF) or visually enhanced lesion scope (Velscope), and optical coherence tomography (OCT) provide assistance in early diagnosis and identification of oral pathophysiologic lesions [11]. The advancement of complete human genome insight, and the numerous potentials of cellular and epigenetic research, can be utilized as prognostic and diagnostic techniques for conducting rapid evaluation and treatment of oral lesions. The molecular diagnostic measures are divided into two types: nucleic acid-associated markers and protein-associated markers. In recent years, several biomarkers have been identified that could be utilized for the prognosis, diagnosis, differential diagnosis, prediction of recurrence, distant metastasis, and chemotherapy or radiotherapy resistance of OC. The biomarkers in OC include up-regulated expression of epidermal growth factor receptor (EGFR), vascular endothelial growth factor receptor (VEGFR), matrix metalloproteinases (MMPs), proliferating cell nuclear antigen (PCNA), Ki-67, Cyclin D1, Cathepsin-d, CD44, cytokeratins, p53 and p16 mutation or expression status, and the prevalence of human papilloma virus (HPV) and its oncogenes [12]. These biomarkers can aid in screening and early detection and prognosis of OC, which can improve treatment outcomes by allowing early intervention. However, these biomarkers are not enough as a primary diagnostic tool, and can only be useful in combination with other diagnostic methods to confirm malignancy and its stage. In addition, these markers are often not organ-specific. Therapeutic intervention by targeting these biomarkers shows limited efficacy and side effects.

## 3. Diverse ncRNAs in OC

Multiple ncRNAs have been identified in association with OC growth, invasion, migration, and therapy resistance. The importance of the ncRNAs in OC is summarized below.

### 3.1. Role of miRNAs in Oral Cancer

The effect of microRNAs (miRNAs) has gained considerable attention in the past few years for regulating the biological processes of multiple malignancies. The miRNAs are 19–25 nucleotide small RNA molecules that mainly interact with the 3′UTR of the target mRNA, and thus regulate gene expression. However, interactions with 5′UTR, promoter, and coding region have also been reported [13]. The miRNA can function as an oncogene or tumor suppressor gene, and the deregulation of miRNAs is seen in many cancers, including OC. Defects in miRNA biogenesis machinery, alterations in miRNA genes or transcriptional regulation, or epigenetic regulations are often associated with the miRNA deregulation mechanism.

With the aim of improving patient survival, numerous miRNAs have been discovered to have roles in the etiology of OC throughout time. The miRNAs associated with tissue, or bio-fluids like blood, and saliva, are found to regulate cancer growth, survival, invasion, metastasis, angiogenesis, chemotherapy, and radiotherapy resistance [14]. Simultaneous suppression of oncomiRs and replacement of tumor suppressor miRNAs are suggested to be an effective approach in designing an OC treatment strategy. OncomiRs like miR-21, miR-155, miR-196, miR-1237, miR-31, miR-455, miR-181, miR-184, miR-134, miR-146, miR-93, miR-372, miR-373, miR-103a-3p, miR-454, miR-654-5p, miR-188-5p, miR-626, miR-4513, miR-944, and miR-650 are found to be up-regulated in OC cell lines and patient samples (Table 1). As stated in Table 1, many of the oncomiRs are found to be associated with OC diagnosis or prognosis, and some are suggested as biomarkers. The functions of the oncomiRs are associated with tumor progression, cell migration, drug resistance, inhibition of apoptosis, and induction of metastasis in OC. On the other hand, miRNAs such as miR-204, miR125b, miR-9, miR-26a/b, miR-491-5p, miR-375, miR-320, miR-218, miR-205, miR-181a, miR-138, miR-124, miR-99a, miR-34, miR-29a, miR-17, etc., function like tumor suppressors and are found to be down-regulated in OC cell lines, and patient samples (Table 1). The miRNAs generally regulate the expression of oncogenes and tumor suppressors to modulate OC progression. For example, the miR-21 is one of the well-studied oncomiRs, and targets many tumor suppressor genes. In OC, the miR-21 interacts and regulates PTEN, RECK, PDCD4, TPM1, DKK2, and CADM1, resulting in induction of proliferation, invasion, and chemoresistance [15,16,17]. The transcription factor AP-1 (activating protein-1) activates miR-21 in various cancers [18]. Exposure of tobacco-smoke-associated nitrosamine 4-(methylnitrosamino)-1-(3-pyridyl)-1-butanone (NNK) induces miR-21 and miR-155 expressions in lung and OC cell lines [19]. The miR-155 is involved in development of many cancers, and can act as both oncogene and tumor suppressor gene. In OC, the miR-155 is up-regulated as oncomiR, and associated with OC progression, metastasis, and drug resistance [20,21,22]. The transforming growth factor β (TGF-β)/Smad4 signaling activates miR-155 expression and promoter activity [23]. The TGF-β, a pleiotropic cytokine, regulates tumor suppression or promoting activity in OC [24]. Overexpression of TGF-β induces metastasis and immune modulation in OC [24]. Another well-studied miRNA is Let-7, which is responsible for cell differentiation in normal cells, and is found to become suppressed in OC [25]. The RNA binding protein Lin28A and B inhibit Let-7 biogenesis by binding at pre-miRNA gene [26]. In addition, lncRNA ROR, lncRNA H19, lncRNA PVT-1, and circ_CPA4, circ_HMCU act as miRNA sponges, and negatively regulate Let-7 expression in cancers [26]. Down-regulation of this miRNA increases expression of Twist and Snail which, in turn, promotes epithelial-to-mesenchymal transition (EMT) and plays a significant role in chemoresistance-to-cisplatin transition, as well as 5-fluorouracil (5-FU) [27]. The miR-124 is found to be deregulated in many cancers, including OC, due to promoter methylation. Overexpression of miR-124 inhibited integrin beta-1 (ITGβ1) and, thus, reduced OC migration [28]. Thus, the difference in the expression profiles gives both oncoMiRs and tumor suppressive miRNAs a role of potent biomarkers or therapeutic targets that might be beneficial to diagnose and treat OC.

### 3.2. Role of Long Non-Coding RNAs in Oral Cancer

Long non coding RNAs (LncRNAs) are more than 200 nucleotides long and they have similar biogenesis to messenger RNAs (mRNAs). They are transcribed by RNA-Polymerase II (Pol II), and undergo polyadenylation, splicing, and 5′-capping [115]. LncRNAs are localized either in the nucleus, cytoplasm, or both. Depending on the localization, they perform a variety of cellular processes by interacting with DNAs, RNAs, and proteins such as epigenetic modification, transcriptional regulation, RNA splicing, mRNA stabilization, translational regulation, sequestration of proteins, protein stabilization, and miRNA sponges [115,116,117,118]. Due to these regulatory functions, lncRNAs are associated with numerous disorders such as diabetes, inflammatory bowel diseases, AIDS, neurodegenerative diseases, different blood-related disorders, as well as cancers [119,120].

The lncRNAs exhibit aberrant expression patterns in OC, and play a significant role in the advancement of the disease [121]. In OC, the lncRNAs may either act as oncogenes or as tumor suppressor genes. The functions of some reported lncRNAs are summarized in Table 2. Oncogenic lncRNAs, such as lncRNAs MALAT1, NEAT1, PVT1, ELDR, DLEU1, HOTAIR, HIFCAR, PCAT1, DANCR, etc., are associated with tumor proliferation, invasion, metastasis, angiogenesis, and drug resistance. For example, high MALAT1 expression is correlated with OC recurrence and metastasis [122]. It has also been demonstrated that MALAT1 promotes cellular proliferation and metastasis via controlling multiple signaling events, including Wnt/β-catenin signaling and PI3K/AKT/mTOR signaling pathways [122]. MALAT-1 is present in circulation, and shows importance as a serum biomarker. The MALAT1 is associated with resistance in multiple drugs, such as cisplatin, 5-fluorouracil, and paclitaxel [122]. Currently, the lncRNA MALAT1 is in OC clinical trial. The MALAT1 promoter contains transcription factors SP1 and SP3 binding sites [123]. Cooperative function of SP1 and SP3 activates MALAT1 expression [123]. In addition, STAT3/ TGF-β signaling axis, hypoxia-inducible factor (HIF)-2α, Yes-associated oncoprotein (YAP)-1, Jumonji C-domain–containing protein (JMJD)-1A, Octamer-binding transcription factor (OCT)-4, chemokine (C-C motif) ligand (CCL)-5, β-catenin, and Lysine-specific demethylase (KDM)-5B are involved in up-regulation of MALAT1 in OC and other cancers [123]. Another lncRNA ELDR is found to be up-regulated in OC cell lines and patient samples [124]. The ELDR inhibits miRNA-7, resulting in stabilization of EGFR. In addition, the ELDR interacts with RNA binding protein Interleukin Enhancer Binding Factor 3 (ILF3), and stabilizes cell cycle gene Cyclin E1. Interestingly, targeted inhibition of ELDR could inhibit in vivo tumor growth in a mouse model [124]. Overexpression of ELDR in normal oral keratinocytes (NOKs) induces cell proliferation and G2/M cell cycle progression through activation of CTCF/FOXM1/AURKA axis showing importance of the lncRNA as one of the OC driver genes [125]. It is not clear why the ELDR is up-regulated in OC; however, around 11% of samples contain the gene amplification in TCGA database (www.cbioportal.org, accessed on 28 June 2023).

On the other hand, the lncRNAs MEG3, GAS5, FENDRR, and PTCSC3 are down-regulated in OC. For example, the down-regulation of the lncRNA GAS5 increases miR-21 expression and helps in proliferation, invasion, EMT, and migration [126]. MEG3, being another tumor-suppressive lncRNA, suppresses miR-421 and the Wnt/β-catenin pathway [127,128]. Down-regulation of the lncRNA helps in the induction of the cell cycle, cell proliferation, metastasis, and suppression of cell apoptosis. Promoter methylation of MEG3 and GAS5 is one of the mechanisms of down-regulation in cancers [129,130]. As stated in Table 2, many lncRNAs are associated with OC diagnosis and prognosis, and are thus suggested to be OC biomarkers and therapeutic targets.

**Table 2:** List of lncRNAs regulating oral cancer.

LncRNAs	Function on Up or Down Regulation	Targets/Associated Pathways	Study Model	Biomarker/Therapy	References
List of up-regulated lncRNAs					
lncRNA DANCR ↑	Promotes proliferation, invasion, and migration, and suppresses apoptosis	*miR-135a-5p/KLF8 axis*, *DANCR/miR-4707-3p/FOXC2 pathway*	Patient samples and cell lines	Biomarker (prognostic and diagnostic), therapeutic target	[131]
lncRNA PCAT1 ↑	Regulation of proliferation, and inhibits apoptosis	*c-Myc-AKT1-p38 MAPK signaling pathways*	Cell lines, patient tissue, and xenograft tumor model	Therapeutic target	[132]
lncRNA PVT1 ↑	Increases metastasis, proliferation, and invasion, enhanced EMT and cancer cell stemness	*Wnt/β-catenin signaling*	Cell lines and xenograft tumor model	Therapeutic target	[133]
lncRNA MALAT1 ↑	Associated with differentiation and clinical staging in TSCC. Correlated with tumor occurrence, development, and prognosis in HNSCC. Chemoresistance in LSCC and OSCC cells	*Wnt/β-catenin in TSCC.* *G2/M in HNSCC.* *PI3K/AKT/mTORsignaling pathway in OSCC.*	Cancer cell lines and tissue and plasma samples	Diagnostic biomarker	[122]
lncRNA-ROR ↑	Regulates cellular differentiation, represses p53	*miR-145*	Clinical specimens	Prognostic biomarker	[134]
lncRNA NORAD ↑	Causes cell proliferation, migration, decreasing apoptosis, sponges miR-577 to enhance TPM4.	*miR-577/TPM4 axis*	OC tissues and cell lines	Therapeutic target	[135]
lncRNA ELDR ↑	Induces cell proliferation, and inhibits miR-7 to regulate EGFR. Regulates Cyclin E1 signaling through ILF3	*ILF3-cyclin E1 signaling*,	Tissue samples, cell lines, and xenograft mouse model	Therapeutic target	[124]
lncRNA HOTAIR ↑	Promotes tumor cell invasion and metastasis, and represses E-cadherin in OSCC	*EZH2*	Tissue, saliva samples and cell lines	Biomarker and therapeutic target	[136]
lncRNA HIFCAR ↑	Modulates the hypoxia signal pathway and contributes to OSCC progression	*hypoxia-inducible factor (HIF-1α)*	OC cell lines and xenograft mice model	Prognostic biomarker	[137]
lncRNA UCA1 ↑	Promotes proliferation and cisplatin resistance, as well as suppressed apoptosis in OSCC cells	*miR-184*	Tissue, saliva samples, cell lines, and xenograft mice model	Therapeutic target	[138]
lncRNA XIST ↑	Regulates miR-29b expression, which induces cell apoptosis through the p53 pathway and promotes tumor growth in in vivo *model*	*miR-29b*	Xenograft model and OC cell lines	Therapeutic target	[139]
lncRNA ARNIL ↑	Promotes proliferation, invasion, and migration	*miR-125a*	Tissues and serum, cell lines, and xenograft mouse model	Biomarker (prognostic)	[140]
lncRNA NEAT1 ↑	Promotes proliferation, migration, and invasion	*miR-365/RGS20*	OC cell lines, tissue, saliva samples, and mice model	Biomarkers and therapeutic target	[141,142]
lncRNA DLEU1 ↑	Promotes proliferation, invasion, and migration	*miR-149/CDK6 axis*	OC cell lines	Therapeutic target	[143,144]
lncRNA AC007271.3 ↑	Promotes proliferation, migration, and invasion, inhibits apoptosis, and induces tumor growth in vivo	*Wnt/β-catenin signaling pathway*, *miR-125b-2-3p/Slug/E-cadherin axis*	OC tissues, saliva, plasma, cell lines, and mice model	Therapeutic target	[145,146]
lncRNA LHFPL3-AS1 ↑	Promotes OSCC growth and cisplatin resistance	*LHFPL3-AS1/miR-362-5p/CHSY1 Pathway*	OC tissues and cell lines	-	[147]
lncRNA 01296 ↑	Promotes proliferation, invasion, and migration	SRSF1 *protein*	Tissue samples, cell lines, and xenograft mice model	Therapeutic target	[148]
lncRNA JPX ↑	Promotes proliferation, invasion, and migration	*miR-944/CDH2 axis*	OC cell lines	Therapeutic target	[149]
lncRNA LINC00974 ↑	Promotes invasion and migration	*miR-122*, *RhoA*	Tissue samples and cell lines	-	[150]
lncRNA PRNCR1 ↑	Promotes proliferation, invasion, and migration	*miR-326/FSCN1 axis*	Cell lines, saliva, and plasma	-	[151]
lncRNA LOLA1 ↑	Promotes migration, invasion, and EMT	*AKT/GSK-3β pathway*	Tissue samples and cell lines	Therapeutic target	[152]
lncRNA MCM3AP-AS1 ↑	Promotes proliferation, migration, and invasion	*miR-204-5p/FOXC1*	Tissues and cells	-	[153]
lncRNA LINC00662 ↑	Increased TNM stage and lymph node metastasis of the patients. Promotes cell growth and metastasis	*miR-144-3p/EZH2 Axis*	Tissues and cell lines	Therapeutic target	[154]
lncRNA HOTTIP ↑	Causes lymph node metastasis. Regulates proliferation, migration, and invasion	*HMGA2-Mediated Wnt/β-Catenin Pathway*	Xenograft model, patient tissue and saliva, and cell lines	Biomarker (diagnosis) and therapeutic target	[155]
lncRNA MIR4435-2HG ↑	Regulates cancer cell behavior. Involved in the promotion of cancer cell proliferation, migration, and invasion	*TGF-β1*	Plasma samples and cell lines	Therapeutic target	[156]
lncRNA GACAT1 ↑	Promotes tumor growth and migration	*miR-149*	Tissue samples and cell lines	Therapeutic target	[157]
lncRNA TSPEAR-AS2 ↑	Promotes tumor cell progression and is associated with advanced TNM staging	*TSPEAR-AS2/miR-487a-3p/PPM1A axis*	Tissues and cell lines	Biomarker and therapeutic strategy	[158]
lncRNA FTH1P3 ↑	Induces cancer cell proliferation, migration, and invasion	*PI3K/Akt/GSK3b/ Wnt/β-catenin*	Tissues and cell lines	Biomarker	[159]
lncRNA PLAC2 ↑	Promotes proliferation and invasion in OSCC cells as well as tumor growth and metastasis in vivo	Downstream *Wnt/β-catenin signaling pathway*	Tissues cell lines, and xenograft mice model	Biomarker (prognosis and therapy)	[160]
lncRNA SNHG20 ↑	Promotes proliferation, migration, and invasion	*miRNA-19b-3p/RAB14 axis*, *miR-29a/DIXDC1/Wntsignaling pathway*	Tissue samples and cell lines	Biomarker (diagnosis), and therapeutic target	[161,162]
lncRNA LINC01137 ↑	Promotes proliferation, invasion, and migration	*miR-22-3p*	Cell lines	Therapeutic target	[163]
lncRNA PSMA3-AS1 ↑	Promotes proliferation, invasion, and migration	*miR-136-5p/FN1 axis*	Patient samples	Prognostic marker	[164]
lncRNA DCST1-AS1 ↑	Causes M2 polarization of tumor-associated macrophages, which thereby promotes tumor malignancy and metastasis	*NF-κB pathway*	OC cell lines and tumor xenograft model	Prognostic indicator	[165]
lncRNA TUG1 ↑	Promotes proliferation, invasion, and migration, prevents apoptosis	*TUG1/miR-593-3p/MAPK axis*	OC cells, tissues, saliva and nude mice model	Therapeutic target and biomarker (diagnosis)	[166]
lncRNA FOXD2-AS1 ↑	Associated with poor pathological grading and prognosis in patients. Promotes proliferation and colony formation as well as regulates the cell cycle signaling pathways	*CDK2*, *CDK4*, *and P21*	OC cell lines	Therapeutic target and biomarker (prognosis)	[167]
lncRNA LINC01234 ↑	Promotes growth, invasiveness, and metastasis	*miR-637/NUPR1 axis*, *miR-433/PAK4 axis*	Tissue samples, cell lines, and nude mice model	Therapeutic target	[168,169]
lncRNA LINC01207 ↑	Promotes proliferation and migration, reduces apoptosis and autophagy of cells	*miR-1301-3p/LDHA axis*	Tissue samples and cell lines	Novel diagnostic and therapeutic target	[170]
lncRNA TTN-AS1 ↑	Promotes cell growth, and migration and restricts apoptosis	*miR-411-3p/NFAT5 axis*	Tissue samples and cells, as well as mice model	Therapeutic target	[171]
lncRNA ZEB1-AS1 ↑	Promotes EMT, cell invasion, and migration. Act as a tumor promoter	*miR-23a*	Patient samples, cell lines, and xenograft mice model	Therapeutic target	[172]
lncRNA H19↑	Promotes proliferation, associated with the TNM staging and nodal invasion	*H19/miR-138/EZH2 axis*	Cell lines, tissue samples, and mice model	Therapeutic target	[173]
lncRNA HCP5 ↑	Facilitates Cell Invasion And EMT	*miR-140-5p/SOX4 axis*	Patient samples and cell lines	Therapeutic target	[174]
lncRNA PTTG3P ↑	Promotes proliferation and migration	*PTTG3P/miR-142-5p/JAG1 axis*	OC cell lines	-	[175]
lncRNA IGF2BP2-AS1 ↑	Promotes cell growth, and migration and restricts apoptosis	*Wnt/β-catenin pathway*	Tissue samples, plasma, and cell lines	Therapeutic target	[176]
lncRNA ADAMTS9-AS2 ↑	Promotes migration and invasion and facilitated metastasis in salivary adenoid cystic carcinoma (SACC)	*Binds with miR-143-3p and activates PI3K/Akt and MEK/Erksignaling*	Tissue samples, cell lines, and xenograft mouse model	Therapeutic target	[177]
lncRNA LTSCCAT ↑	Promotes EMT and promotes invasion and metastasis in both in vivo and in vitro	*miR-103a-2-5p/SMYD3/TWIST1 axis*	Tissue samples, cell lines and xenograft tumor model	Therapeutic target	[178]
lncRNA RP11-284F21.9 ↑	Promotes proliferation, invasion, and migration	*miR-383-5p/MAL2 axis*	Tissue samples and cancer cell lines	Therapeutic target	[179]
lncRNA WWTR1-AS1 ↑	Associated with larger tumor size, cervical node metastasis, and poor prognosis	*WWTR1-AS1/WWTR1 axis*	Cell lines	Biomarkers with prognostic significance	[180]
LINC00668 ↑	Facilitate VEGFA expression, and promotes tumor growth	*miR-297/VEGFA axis*	OC cell lines and tissues	Biomarker (diagnosis) and therapeutic target	[181]
lncRNA HNF1A-AS1 ↑	Promotes OSCC progression	*Notch signaling pathway*	Tissue samples and cell lines	Therapeutic target	[182]
lncRNA MINCR ↑	Causes proliferation and migration	*Wnt/β-catenin pathway*	Tissue samples and cancer cell lines	Prognostic biomarker and therapeutic target	[183]
lncRNA LACAT1 ↑	Promotes malignant progression	*microRNA-4301*	Tissue samples and cancer cell lines	-	[184]
lncRNA CASC9 ↑	Enhances tumor progression via suppression of autophagy-mediated cell apoptosis	*AKT/mTOR pathway*	Tissue samples, cell lines, and mice model	Biomarker (diagnosis and prognosis)	[185]
lncRNA SNHG26 ↑	Promotes TSCC growth, metastasis, and cisplatin resistance	*PGK1/Akt/mTOR*	Tissue samples, cell lies, and xenograft mice model	Therapeutic target and biomarker (diagnosis)	[186]
lncRNA PART1 ↑	Promotes proliferation and inhibits apoptosis	*EZH2*	Tissue samples, cell lines, and mice model	Diagnosis biomarker and a novel therapeutic target	[187]
lncRNA LINC00152 ↑	Induces tumor progression, and is associated with tumor size, invasion of muscles of the tongue, lymph node metastasis, and recurrence as well	*-*	Tissue samples	Biomarker (diagnosis and prognosis)	[188]
lncRNA HOXA-AS2 ↑	Causes OC cell proliferation and promotes tumor growth in vivo	*miR-567/CDK8*	Tissue and plasma samples, cell lines, and xenograft model	Biomarker (prognostic) and therapeutic target	[189]
lncRNA DNM3OS ↑	Modulates cell viability and migration	*DNM3OS/miR-204-5p/HIP1 axis*	Clinical samples and OC cell lines	Therapeutic target	[190]
lncRNA SNHG1 ↑	Leads to the proliferation of cancer cells	*miR-421/HMGB2 axis*	Cancer cell lines	Therapeutic target	[191]
lncRNA HOXA10-AS ↑	Promotes OC growth, and metastasis.	*TP63* mRNA	Xenograft model and cancer cell lines	Therapeutic target	[192]
lncRNA BBOX1 ↑	Encourages proliferation and migration, and suppresses apoptosis	*miR-3940-3p/laminin subunit gamma 2 axis*	Tissue, saliva and cell lines	Therapeutic target	[193]
lncRNA IFITM4P ↑	Induces cell proliferation and enhanced immune escape	*PD-L1*	Tissue samples, cell lines, and xenografted tumors	Therapeutic target	[194]
lncRNA CACS15 ↑	Promotes proliferation and reduces expression of lncRNA MEG3	*lncRNA MEG3*	Tissue and plasma samples	Diagnostic biomarker	[195]
lncRNA LINC00963 ↑	Promotes cancer stemness, increases cancer aggressiveness, and reduces chemosensitivity	*ABCB5*	Tissue samples, cancer cell lines, and xenograft nude mice model	Therapeutic target	[196]
lncRNA OIP5-AS1 ↑	Enhances cancer stemness, and is associated with poor clinical outcome	*-*	Clinical specimens	-	[197]
lncRNA KCNQ1OT1 ↑	Increases cisplatin resistance, regulates proliferation and metastasis of cisplatin-resistant TSCC	*KCNQ1OT1/miR-124-3p/TRIM14 axis*	Cisplatin-resistant TSCC samples and TSCC cell lines	-	[198]
lncRNA BLACAT1 ↑	Regulates viability, and causes migration and invasion of cells	*miR-142-5p*	OC cell lines	Therapeutic target	[199]
lncRNA AFAP1-AS1 ↑	Encourages tumor proliferation and indicates a poor prognosis	*CCNA2*	OC cell lines, xenograft tumor model	Unfavorable biomarker, therapeutic target	[200]
lncRNA FAL1 ↑	Causes proliferation and develops OSCC	*microRNA-761/CRKL pathway*	Tissues and cell lines	Therapeutic target	[201]
lncRNA HOXA11-AS ↑	Promotes proliferation, and facilitates CDDP-resistance	*miR-214-3p/PIM1*	Clinical tissue specimens, cell lines, and xenograft mice model	Therapeutic target	[202]
lncRNA FEZF1-AS1 ↑	Promote the malignant progression of OSCC	*miR-196a*	Patient samples and OSCC cell lines	-	[203]
lncRNA SNHG6 ↑	Improves cell viability, proliferation, and EMT. Inhibits apoptosis	*β-catenin and E-cadherin*	Tca1183 cells	-	[204]
lncRNA HOXC13-AS ↑	Induces proliferation, migration, and EMT	*miR-378g/HOXC13 axis*	Patient samples and cell lines	Therapeutic target	[205]
lncRNA ORAOV1-B ↑	Induces invasion, migration, and metastasis	*Binds to Hsp90 and activates the NF-κB-TNFα loop.*	OC cell lines	Therapeutic target	[206]
lncRNA LINC00319 ↑	Induces proliferation, metastasis, EMT, invasion, and angiogenesis	*miR-199a-5p/FZD4 axis*	Cancer cells and tissues	Therapeutic target	[207]
lncRNA SLC16A1-AS1 ↑	Promotes proliferation and accelerates cell cycle	*SLC16A1-AS1/CCND1 (requires further elucidation)*	OC cell lines and patient tissue and plasma samples	Therapeutic target and diagnostic indicator	[208]
lncRNA RP5-916L7.2 ↑	Induces proliferation and represses apoptosis	*miR-328 and miR-939*	Patient samples and OC cell lines	-	[209]
lncRNA LINC00284 ↑	Causes cell proliferation and migration	*miR-211-3p/MAFG axis*	Patient samples and cell lines	Biomarker	[210]
lncRNA LINC00958 ↑	Promotes proliferation, migration, EMT and retards apoptosis	*miR-627-5p/YBX2 axis*	Tissue, saliva, and cell lines	Therapeutic target and biomarker (prognostic)	[211]
lncRNA LEF1-AS1 ↑	Increases cell survival, proliferation, and migration. Retards cell apoptosis	*LATS1*	Plasma samples and cell lines	Therapeutic target and biomarker	[212]
lncRNA FOXC2-AS1 ↑	Improves proliferation, invasion, migration, and EMT and regulates the cell cycle	*miR-6868-5p/E2F3 axis*	Patient samples and cell lines	Therapeutic target	[213]
lncRNA LINC01116 ↑	Causes migration and invasion	*LINC01116/miR-9/MMP1 axis*	Patient samples and cell lines	Therapeutic target	[214]
List of down regulated lncRNAs					
lncRNA MORT ↓	Low expression is associated with increased proliferation and poor survival	*ROCK1*	Cell lines and patient samples	Therapeutic target	[215]
lncRNA AC012456.4 ↓	Significantly associated with tumor staging and survival rates for patients	*JAK-STAT and MAPK signaling pathways*	Cell lines and patient samples	Diagnostic, therapeutic and prognostic biomarker	[216]
lncRNA MEG3 ↓	Down-regulation alleviates the aggressiveness of cancer, and is associated with poor prognosis. Induces tumor growth by promotion of cell proliferation and metastasis, induced cell cycle and suppressed cell apoptosis	*miR-421*, *Wnt/β-catenin pathway*	OC cell lines and patient samples	-	[127]
lncRNA HCG11 ↓	Enhances OSCC proliferation, increases G1/S transition and Ki67 levels	*miR-455-5p*	OC cell lines	Therapeutic target	[217]
lncRNA SCIRT ↓	Inhibits cancer cell apoptosis	*miR-221*	Patient samples	Biomarker	[218]
lncRNA C5orf66-AS1 ↓	Induces proliferation, invasion, and migration and inhibits apoptosis	*CYC1*	Clinical specimens and cell culture	Therapeutic target	[219]
lncRNA PTCSC3 ↓	Promotes cancer cell proliferation and invasion	*-*	Tissues and cell lines	Therapeutic target	[220]
lncRNA GAS5 ↓	Promotes proliferation, invasion, EMT, and migration	*miR-21/PTEN axis*	Tissue, serum, and cancer cell lines	Therapeutic target	[126]
lncRNA FENDRR ↓	Fails to inhibit angiogenesis of OSCC	*PI3K/AKT pathway*	Cell lines and patient samples	Therapeutic target	[221]

Up arrows ↑ indicate upregulation and down arrows ↓ indicate downregulation.

### 3.3. Role of Circular RNAs (circRNA) in Oral Cancer

CircRNAs are closed-loop, extremely stable ncRNA molecules with no 3′ or 5′ ends and a poly (A) tail. They belong to the lncRNA kingdom, and have a higher half-life compared to linear RNAs [222,223]. Back-splicing and exon skipping of pre-mRNAs are the two processes by which circRNAs are produced. The transcription process is carried out via RNA pol II [224]. These RNA structures have the ability to withstand exonucleolytic breakdown by RNase R. It has been seen that 80% of circRNAs are present in the cytoplasm. However, the presence of circRNAs in the nucleus makes the control of gene expression possible [225].

Due to their closed-loop orientation, tissue specificity, high stability, and conservation, they serve as significant biomarkers for several diseases. These RNA molecules perform several regulatory functions, such as miRNA sponging, direct protein binding, and certain circRNAs are even translated into proteins [226,227]. Numerous studies have revealed that the majority of aberrantly expressed circRNAs play a significant role in controlling the progression of cancer by influencing a number of cancer hallmarks. Proliferative signaling, encouraging tumor and antitumor immunity, triggering angiogenesis, promoting invasion, metastasis, and deregulating cellular energetics are some functions that are associated with an aberrant circRNA profile [228].

Research on circRNA in OC has increased in recent years, and it has been discovered that these RNA molecules have a significant influence on the development, management, and prognosis of OC [229]. Like miRNA and lncRNA, these RNA molecules also play a role as both oncogenes and tumor suppressors in OC. For example, circ_0002185, circ_PVT1, circ_100290, circ_0001742, circ_HIPK3, circ_0001971, circ_DOCK1, circ_FLNA, circ_GOLPH3, circ_CLK3, circ_CDR1, circ_0014359, circ_LPAR3, circ_SEPT9, etc., are up-regulated, whereas circ_0000140, circ-PKD2, circ_0005379, circ_0004491, circ_SPATA6, circ_0086414, circ_0008309, circGDI2, circ_0007059, etc., are down-regulated in OC (Table 3). The mechanism of differential expression of the circRNA in OC is not clear. The oncogenic circular RNA Circ_100290 acts as competing endogenous RNA (ceRNA), and inhibits miR-378a mediated suppression of glucose transporter GLUT1, resulting in the induction of glycolysis and cell growth [230]. The circ_PVT1 is derived from exon 3 of the oncogene of lncRNA PVT1 [231]. Recently, it was discovered that the mutant p53/ YAP/ TEAD transcription-competent complex is responsible for the up-regulation of circ_PVT1 in head and neck squamous cancer [231]. Through sponging miR-125b, Circ_PVT1 worked as a competitive endogenous RNA (ceRNA) to induce STAT3 signaling and cell proliferation [231]. Another circular RNA, called Circ_CDR1, has been stated to encourage autophagy under the hypoxic condition to enhance cell survival in OC, via control of the AKT/ERK-1/2/mTOR signaling pathway [232]. On the contrary, a study showed the association between suppressed expression of circ_0007059 and OC, via regulation of the AKT/mTOR pathway [233]. A high throughput sequencing study of OC samples identified significant down-regulation of circ_0005379 as compared to the adjacent normal tissues [234]. Up-regulation of circ_0005379 enhances cetuximab sensitivity, efficiently reduces OC proliferation, migration, invasion, and angiogenesis in vitro, and slows tumor growth in nude mice via inhibiting EGFR signaling [234]. All these studies suggest that these RNA molecules regulate OC via control over the major signaling pathways like MAPK, WNT/β-catenin, Notch, VEGF, and PI3K/AKT in OC [235]. The aggressive trait of OC was found to be linked to circ_PKD2 down-regulation. Overexpression of circ_PKD2 induces cell cycle arrest and apoptosis, and inhibited proliferation, migration, and invasion of OC through inhibiting miR-204-3p [236]. All these studies indicate the potential role of circRNA in OC. Table 3 enlists several other circRNAs whose aberrant expression level regulates OC.

### 3.4. Role of Small Nucleolar RNA (SnoRNA) in Oral Cancer

SnoRNAs are one of the many classes of non-coding RNA molecules present in the body. There are around 300 snoRNA sequences identified in the human genome. Although, snoRNAs are small in size, they are present in large quantities within the nucleus of cells [269]. Most snoRNAs are expressed in the intron of both coding and non-coding genes, while the remaining gets transcribed by RNA polymerase II (RNA pol II). Splicing, debranching, and co-transcription are the stages involved in their biogenesis. They play diverse roles in the development of ribosomal, small nuclear, and other pre-mRNA molecules via endonucleolytic disintegration and post-transcriptional regulation [269]. They also have the ability to control gene expressions by modifying and splicing mRNA. The snoRNAs form small nucleolar ribonucleoprotein complexes (snoRNP complexes) by binding to protein molecules, which then leads to the modification of rRNA bases [269].

SnoRNAs have roles in a variety of pathological and physiological processes. Studies have shown that snoRNAs control tumor growth, invasion, and metastasis, as well as cell death during the carcinogenesis process. More importantly, snoRNAs play a significant role in the development of OC tumors. Today, the differential expression of snoRNAs in OCs leads to the possibility of them being used as diagnostic and prognostic biomarkers [270]. However, the mechanism of differential expression of snoRNA in OC is not known clearly. Alteration in snoRNA biogenesis and post-transcriptional regulation may be involved in differential expression of different snoRNAs in OC. An in silico investigation using RNA seq data of 567 samples from the TCGA head and neck cancer cohort could identify 113 snoRNAs using *p*  < 0 .05 as the cut-off [271]. The top significantly modulated snoRNAs were associated with DNA template regulation, RNA editing, regulation of cell proliferation, adhesion, invasion, metastasis, PI3K-AKT signaling, EMT, and angiogenesis pathways. Further analysis with the top five snoRNAs (SNORD114-17: ENSG00000201569, SNORA36B: ENSG00000222370, SNORD78: ENSG00000212378, U3: ENSG00000212182, and U3: ENSG00000212195) showed association with patient survival, indicating the importance of snoRNAs in disease progression and as biomarkers in OC [271]. A microarray analysis from eight OC samples identified 16 significantly modulated snoRNAs as compared to control samples; among them, 15 were significantly down-regulated and associated with patient survival [270]. The SNHG3, a snoRNA that gets up-regulated in OC patients, induces migration and cell proliferation of oral squamous cells. It targets the nuclear transcription factor-Y subunit gamma (NFYC) via the SNHG3/ miR-2682-5p axis and functions as a biomarker [272,273]. SnoRNA SNHG15 also gets overexpressed in OC cell lines, and facilitates the malignant behaviors of OC via miR-188-5p/ DAAM1 as a target [274]. Thus, snoRNAs play a role in assisting tumor growth in OC. Further studies can increase their significance in cancer therapy in the future. Table 4 enlists expressions and functions of some snoRNAs in OC.

### 3.5. Role of piRNAs in Oral Cancer

PIWI-interacting RNAs (piRNAs) are a subclass of ncRNAs that can be divided into three primary categories: transposon-derived piRNAs, mRNA-derived piRNAs, and lncRNA-derived piRNAs. They are 24–31 nucleotides long, have 5′-end uridine or 10th position adenosine bias, and lack proper secondary structural features [278]. The piRNAs, composed of an array of different nucleotide sequences, are single-stranded ncRNAs that interact with P-element-induced wimpy testis (PIWI) proteins [279]. They are the largest group of ncRNAs, and are multi-functional. PiRNAs are instrumental in genome rearrangement, spermiogenesis, protein regulation, transposon silencing, epigenetic regulation, and germ stem-cell maintenance by binding to PIWI proteins to make a piRNA/PIWI complex [280].

The piRNAs are mostly known to be expressed in germ cells; however, their existence is also observed in cancer cells. Therefore, the question of employing these RNA molecules as a prognostic marker or therapeutic target arises. Current research has given evidence of the piRNAs/PIWI complex being used for the occurrence, development, metastasis, and recurrence of breast cancer [281] and lung cancer [282]. Some piRNAs have been found to have a role in the development of OC, and can be a potential biomarker or therapeutic target for OC in the future [283]. In the OC mouse model, piR354, piR415, piR832, and piR1584, have been found to interact with mRNA molecules [284]. Longer survival of patients with head and neck cancer is associated with low levels of piR-58510 and piR-35373 [285]. It is observed that genes like GALNT6, SPEDF, and MYBL2 that are paired with piRNAs are responsible for the suppression or progression of several OC tumors [284]. An in silico analysis using the RNA sequencing data of 455 head and neck cancer samples, and 43 matched non-tumors from The Cancer Genome Atlas (TCGA), showed a total of 305 piRNAs in both tumor and non-tumor tissues [286]. Among a total of 247 significantly altered genes, 25 piRNAs were exclusively expressed in non-tumor samples, and 87 were only expressed in tumors. The significantly up-regulated piRNAs, including the topmost gene FR140858, were associated with poor patient survival. This indicates the importance of piRNAs in OC as diagnostic and prognostic biomarkers [286]. Another study identified a panel of 30 piRNAs in 77 HPV positive head and neck cancer samples from the TCGA RNA seq data [287]. Simultaneous validation in cell lines further reported key piRNAs NONHSAT077364, NONHSAT102574, and NONHSAT128479 in HPV associated head and neck cancer development. Based on analysis of the tongue cancer GEO database (GSE196674 and GSE196688), 406 differentially expressed piRNAs were identified [288]. Further investigation identified a down-regulated piRNA: piR-33422 and its association with mevalonate/ cholesterol-pathway-related gene FDFT1 in tongue cancer. Using TCGA RNA seq data of 256 smoking-related head and neck cancer samples, a panel of 13 piRNAs were identified [289]. Among them, NONHSAT123636 and NONHSAT113708 were found to be associated with tumor stage, NONHSAT067200 with patient survival, and 6 other piRNAs with TP53 mutation and 3q26, 8q24, and 11q13 amplification. Further studies are needed to know the regulation of their expression and functional mechanism of piRNAs in OC.

## 4. NcRNAs in Oral Cancer Progression

**Figure 2:**
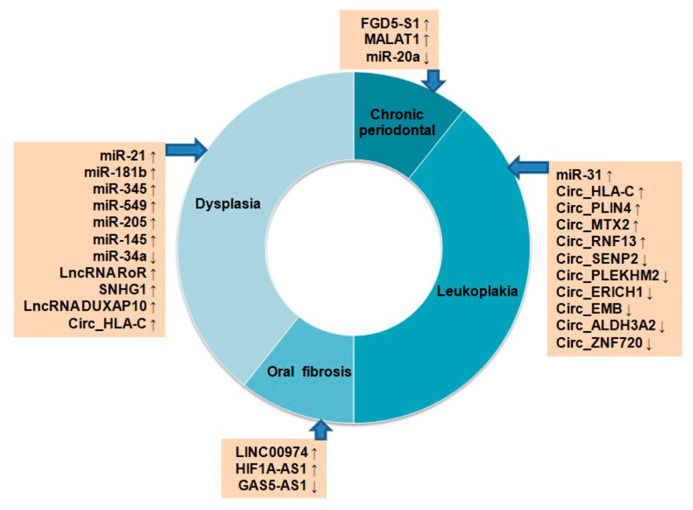
Differential expression of non-coding RNAs in oral pre-cancerous lesions. Up ↑ and down ↓ arrows indicate their up- or down-regulation status.

OSCC or OC is a multistep process originating from epithelial cells by progressive accumulation of genetic and epigenetic alterations. The histological changes that occur during the carcinogenesis begin with atypical squamous cell hyperplasia to carcinoma in situ (CIS) through stages of dysplasia [290]. The molecular events associated with alterations in different protein coding genes during the development of OC are extensively studied [290]. However, the role of ncRNAs is not well-studied in this regard. Few studies have reported the potential modulation of miRNAs, lncRNAs, and circRNAs in premalignant lesions, including oral leukoplakia (LK), oral lichen planus (OLP), oral submucous fibrosis (OSF), and oral dysplasia, with respect to OC (Figure 2).

Up-regulation of miR-7, miR-31, miR-1293, and down-regulation of miR-133a, miR-204 and miR-206 were reported in OC samples. Among these miRNAs, significantly high expressions of miR-31 and down-regulation of its target gene C-X-C motif chemokine ligand 12 (CXCL12) were seen in LK and OLP tissues, suggesting their importance in OC progression from pre-cancerous stages [291]. The miR-21, miR-181b, miR-345, miR-549 and miR-205, were found to be overexpressed both in progressive dysplasia and OC [292]. Another study reported up-regulation of miR-145, lncRNA RoR, and SNHG1 and down-regulation of miR-34a from low-grade to high-grade dysplasia and, finally, to OC during carcinogenesis [293]. The lncRNA FGD5-AS1 inhibits NF-kB signaling, and gets down-regulated in chronic periodontal samples as compared to the healthy tissues [294]. On the other hand, lncRNA MALAT1 was found to be up-regulated in periodontal samples, and induced inflammation through TLR4 by targeting miR-20a [294]. In OSF, up-regulation of lncRNA LINC00974, HIF1A-AS1, and down-regulation of GAS5-AS1 were seen during the development of OSF [294]. An in silico study examined microarray data of 167 OC, 17 dysplasia, and 45 normal oral tissues from the GEO database for expression analysis of lncRNAs [295]. Among these groups, 200 lncRNAs were found to be common in three groups, and 1206 genes are common in OC vs. dysplasia groups. The differentially expressed genes (DEGs) identified among the three groups were found to regulate OC development through PI3K–Akt signaling and NF-kB signaling. Among the DEGs, lncRNA DUXAP10 is relatively new, and associated with the progression of OC development [295]. A high throughput sequencing study identified 366 significantly modulated circRNAs, including 65 up-regulated and 301 down-regulated in LK tissues as compared to the normal mucosa indicating their importance in OC development [296]. Some of the top significantly up-regulated circRNAs were Circ_HLA-C, Circ_PLIN4, Circ_MTX2, Circ_RNF13, and the down-regulated ones were Circ_SENP2, Circ_PLEKHM2, Circ_ERICH1, Circ_EMB, Circ_ALDH3A2, and Circ_ZNF720. The Circ_HLA-C showed stage-wise up-regulation from mild to severe dysplasia [296]. All these studies indicate the importance of ncRNAs in OC development from precancerous lesions to the most aggressive form; however, more mechanistic investigation is needed in this regard.

## 5. NcRNAs in Body Fluid and Exosomes of Oral Cancer as Diagnostic Markers

Even though there has been a lot of improvement in the treatment of OC in the last few years, the prognosis for OC remains poor. Involvement of extracellular vesicles or exosomes in biofluids, like blood and saliva, is seen in disease progression, cellular communication, and metastasis of many cancer types, including OC [297,298,299]. Moreover, studies have revealed that biomarkers in the blood are more intriguing, due to their low invasiveness and increased stability. In a recent study, aberrant expressions of 18 different circulating miRNAs have been discovered that possess a direct association with a poor prognosis for head and neck cancer [300]. Salivary exosomal miRNA-1307-5p was seen as a potent prognostic indicator for oral malignancies, as it has shown the ability to indicate poor prognosis as well as poor patient outcomes [301]. Increased levels of lncRNA TIRY derived from exosomes have been found to reduce miR-14 expression levels which, in turn, enhances OC progression and metastasis [302]. In another study, Li et al., have mentioned two lncRNAs, namely MAGI2-AS3 and CCDC144NL-AS1, derived from serum exosomes that encourage cellular proliferation, migration, and invasion in OC, via regulating the PI3K-AKT-mTOR pathway [303]. Several circRNAs have also been found to have their role as potent biomarkers in OC. A high level of circ_0000199 was seen in circulating exosomes of OC patients, which was found to be associated with poor survival outcomes, proving itself as a potent biomarker for OC [247]. The circ_0001874 and circ_0001971 derived from the saliva of patients were up-regulated in OC and identified as a potential diagnostic biomarker [250]. Table 5 enlists the expression of some of the ncRNAs, which are derived from exosomes, blood, serum/ plasma, and saliva samples, and have significance as biomarkers.

## 6. Non-Coding RNA in Oral Cancer Clinical Trials

The utilization of cancer-specific ncRNAs as biomarkers and therapeutic targets could aid in tailoring treatment options to specific patients or patient subgroups. In contrast to the typical tumor markers being used in latest medical applications, such as protein biomarkers and metabolic products, growing research indicates that ncRNAs could be ideal agents in cancer diagnosis and therapy. This is because the ncRNAs target multiple druggable and non-druggable targets and signaling events at a time. Further, they have tissue specificity, distinct RNA attributes of rapid detection, extra tissue-associated activity, and a far more stable structure [325]. Several clinical trials have been conducted using ncRNAs, especially using miRNAs as diagnostic and therapeutic biomarkers [326]. Through head-on targeting of gene sequences using antisense oligonucleotides (ASOs) and siRNA-associated therapeutic applications, the most sophisticated and concise therapeutic efforts at RNA screening have been achieved to date [326]. The lncRNA H19 promoter sequence has been introduced with the coding sequence of diphtheria toxin in BC-819 plasmid in clinical trials of the bladder, pancreatic and ovarian cancer [115,327]. The lncRNA HOTAIR and CCAT1 (ClinicalTrials.gov) are in clinical trials for thyroid and colorectal cancer diagnostic biomarker studies, whereas inhibitors of LINC01212 and lncMyoD are used as therapeutic markers for melanoma (US2016271163) and sarcoma therapy (WO2015020960) [115,327]. All these studies indicate possible clinical implications of ncRNAs in cancer diagnosis and therapy. A clinical study has been initiated to evaluate the sensitivity and specificity of miRNA-412 and miR-512 in extracellular vesicles from saliva in the malignant progression of OC (ClinicalTrials.gov Identifier: NCT04913545). Another trial has recently been initiated to identify diagnostic and prognostic miRNA biomarkers from blood, saliva, and tissue samples of head and neck cancer (ClinicalTrials.gov Identifier: NCT04305366). The diagnostic and therapeutic importance of lncRNA MALAT1 and its target miR-124 has been studied in saliva samples from OC patients (ClinicalTrials.gov Identifier: NCT05708209). A randomized phase II study has been initiated to identify salivary and plasma miRNAs of head and neck cancer patients, and monitor their change during the dietary intervention (ClinicalTrials.gov Identifier: NCT02869399). Two studies have been performed to assess the correlation of head–neck cancer immunotherapy with blood and plasma miRNAs profiles (ClinicalTrials.gov Identifier: NCT03843515 and NCT04453046). Low EGFR-AS1 lncRNA expression is determined as companion diagnostic biomarker of OC, and a phase II clinical study is recently ongoing to evaluate therapeutic efficacy of a certain drug of EGFR-associated advanced OC with low-EGFR-AS1 OC patients (ClinicalTrials.gov Identifier: NCT04946968). Outcomes of the study may indicate the diagnostic and therapeutic importance of EGFR-AS1 in OC treatment.

## 7. Future Perspectives

Specific ncRNA expression changes have been linked to disease progression and poor outcomes in OC patients. Moreover, the ncRNAs show a promising role in regulating multiple signaling pathways in the modulation of OC progression, invasion, and metastasis. Nevertheless, such conclusions still have to be transformed into medical settings for OC patient populations. This could be attributable to a lack of scientific and technological evaluation research in pre-clinical systems, a dearth of prospective cohort clinical studies, inadequate standardization of the work process to extract and enhance ncRNA, and an absence of standardization targets. Since OC is linked to both behavioral risk factors and genetic predisposition, investigations must account for population-level variability; thus, substantial research regarding patient cohort study from various global locations are required to confirm these benchmarks. In short, the field of ncRNA research in OC is rapidly advancing, and holds great promise for improving our understanding of the disease and developing new strategies for diagnosis, treatment, and prevention. However, there are still many unknowns, and much more research is needed to fully understand the complex roles that ncRNAs play in OC. There are many ncRNAs that are yet to be explored. Many modified ncRNAs are designed to improve their stability and target specific efficacy. Thus, studies in pre-clinical and clinical systems, modifications to increase in-vivo stability and organ-specific targeting, extensive validation, and follow-up studies are needed to fully realize the potential of ncRNAs in OC. A rapid detection kit to identify ncRNAs in blood or saliva samples would greatly improve the diagnostic accuracy of OC. Thus, the acquired information in these areas will pave the way for more important healthcare, prognostic, and treatment options for the management of OC in the near future.

## 8. Conclusions

In summary, the review describes the substantial role of both quantitative and qualitative alterations of different ncRNAs (such as miRNA, lncRNA, circRNA, snoRNA, and piRNA) in the development of OC. The ncRNAs target multiple signaling molecules at a time, and regulate cell proliferation, survival, angiogenesis, metastasis and drug resistance. Studies have revealed that the high death rate and morbidity in OC are correlated with the complexity of conducting a quick diagnosis and appropriate management. Therefore, a timely diagnosis can prove to be crucial for controlling potential invasion and metastasis of oral premalignant conditions, and can also increase the overall life expectancies of patients. Keeping this in mind, a lot of emphasis is being given to the regulative function of different ncRNA profiles, as they demonstrate great promise in identifying OC lesions. Large numbers of ncRNAs like miRNAs, lncRNAs, and circRNAs obtained from exosomes or blood, serum, and saliva unveil their implicit role as non-invasive diagnostic and prognostic biomarkers for OC. Some clinical studies have been initiated to identify blood or saliva miRNA biomarkers in OC patients. miR-412, miR-512, miR-124, lncRNAs MALAT-1, and EGFR-AS1 are currently in OC clinical trials as diagnostic and therapeutic biomarkers, and the outcomes are yet to be received. Recent research on the role of other ncRNA molecules, like snoRNAs and piRNAs, behind the cause of OC development also makes them potential contenders for early diagnosis tools. However, in order to properly integrate liquid biopsy tests into the clinical practice in OC diagnosis, more in-depth pre-clinical research, as well as experimental studies with large cohorts, would indeed be required to confirm these findings and the effectiveness of these biomarkers in OC.

## Figures and Tables

**Table 1:** List of miRNAs regulating oral cancer.

miRNA Name	Function on Up or Down Regulation	Target Genes/Associated Pathways	Study Model	Bio-Marker/Therapy	References
List of Up-regulated miRNAs					
miR-21 ↑	Associated with cell growth, cell proliferation, and cell cycle progression, as well as inhibits apoptosis	*DKK2*, *PTEN*, *PDCD4*, *CADM1*, *RECK*	Patient tissue, saliva, and plasma samples, cell lines	Biomarker (diagnosis and prognosis) and therapeutic target	[15,22,29,30]
miR-155 ↑	Enhances cell proliferation in patients and confers cisplatin chemoresistance via EMT	*FOXO3a*	Patient tissue samples and blood exosome	Prognostic biomarker	[20,21,22]
miR-196a ↑	Promotes cell migration, invasion, and lymph node metastasis	*SPRR2C*, *ANXA1*, *S100A9*, *NME4-JNK-TIMP1-MMP signaling pathway*, *MAMDC2*,	Patient tissue, saliva, plasma samples, and OC cell lines	Biomarker	[30,31,32]
miR-1237 ↑	Causes cellular progression	*FAM69C*, *PACS2*, *SPOP*	Patient tissue and plasma samples	Biomarker (diagnosis and prognosis)	[30]
miR-31 ↑	Increases oxidative stress in OSCC and enriches the oncogenicity and stemness of HNSCC. Regulates the reprogramming of lipid metabolism and enhances cell migration in OSCC	*Ku80*, *FIH*, *ARID1A*, *SIRT3*, *ACOX1*	OC cell lines, tissue, plasma and saliva samples, and transgenic mice	Biomarker	[33,34,35,36,37]
miR-455 ↑	Increases proliferation and anchorage-independent growth of OSCC cell	*UBE2B*	OC Cells, tissue and plasma samples, and xenograft mice model	Biomarker (prognosis)	[38]
miR-181 ↑	Increases cell migration and invasion and may enhance lymph-node metastasis	*-*	Patient tissue sample and plasma	Biomarker	[39]
miR-184 ↑	Increased cell proliferation rate and suppressed apoptosis	*c-MYC*	Tumor tissue, cell lines, and plasma	Biomarker (diagnosis)	[40]
miR-134 ↑	Increases migration and reduces E-cadherin expression in OSCC cell lines. Enhances metastatic potential of OSCC xenografts	*WWOX*, *PDCD7*	Tissue and plasma samples and OSCC cell lines	Biomarker	[41,42]
miR-146a ↑	Increases the tumorigenicity and metastasis	*IRAK1*, *TRAF6 and NUMB*	Tissue, plasma and saliva samples, cell lines, and xenograft tumor model	Biomarker (diagnosis) and therapeutic target	[43]
miR-93 ↑	Promotes metastasis, correlated with poor prognosis	*--*	OC tissue, saliva samples and cell lines	Prognostic marker and therapeutic target	[44]
miR-10b ↑	Role in cancer stemness and facilitates metastatic colonization	*DIAPH2*	Patient tissue and saliva samples and cell lines	Diagnostic marker and therapeutic target	[45]
miR-372 ↑	Enhanced drug resistance and migration. Also associated with lymph node metastasis and poor prognosis	*p62*, *ZBTB7A*	Cell lines, patient tissue and saliva samples, and xenograft tumor model	Diagnostic and prognostic marker	[45,46,47]
miR-373 ↑	Enhances proliferation and metastasis	*SPOP*	OC samples and cell lines	Therapeutic target	[48]
miR-103a-3p ↑	Induces proliferation and restricts cell apoptosis	*RCAN1*	OC cell lines, tumor tissues, saliva, plasma, and xenograft tumor model	Diagnostic marker and therapeutic target	[49]
miR-454 ↑	Involved in proliferation and colony formation, as well as inducing invasion and migration	*NR3C2*	OC cell lines, tissues and saliva samples	Potential therapeutic direction for diagnosis and prognosis	[50]
miR-654-5p ↑	Facilitates EMT, promotes cellular proliferation, tumor metastasis, and chemoresistance	*GRAP*, *Ras/MAPK Signaling*	OC cell lines, patient tissues, plasma, and xenograft tumor model	Biomarker for the clinical diagnosis and prognosis	[51]
miR-188-5p ↑	Causes cell migration	*FOXN2*	Cell lines	Potential prognostic marker and therapeutic target	[52]
miR-626 ↑	Promotes cancer cell proliferation, colony formation, migration, and G0/G1-to-S phase transition	*NFIB*	Patient tissue, plasma, cell lines, and xenograft tumor model	Therapeutic target	[53]
miR-4513 ↑	Causes proliferation and apoptosis	*CXCL17*	Saliva, plasma, and OC cell lines	Therapeutic target	[54]
miR-944 ↑	Induces pro-inflammation cytokines secretion, migration, and invasion	CISH/STAT regulation	OC cell lines, tissue and saliva samples	--	[55]
miR-211 ↑	Causes proliferation, migration, invasion, and metastasis	*BIN1*, *TCF12*, *TGFBRII*, *c-MYC*	Tissue samples, cell lines, and xenograft tumor model	Therapeutic target and prognostic marker	[56,57,58,59]
miR-223 ↑	Promotes OSCC proliferation and migration	FBXW7	Tissue, saliva samples and cell lines	Therapeutic target	[60]
miR-221 ↑	Regulates proliferation and invasion and inhibits apoptosis	*PTEN*	OC cell lines	Biomarker (diagnosis)	[61,62]
miR-222 ↑	Causes proliferation, metastasis, and invasion and decreases apoptosis	*PTEN*, *ABCG2*, *PUMA*	Tumor tissue, saliva samples, and cell lines	Biomarker (diagnosis) and therapeutic target	[61,62,63,64]
Mir-5100 ↑	Causes proliferation, migration, and invasion	SCAI	OC cell lines	Therapeutic target	[65]
miR-382-5p ↑	Induced cell migration and invasion	-	Patients tissue and saliva samples, and TSCC cell lines	Therapeutic target	[66]
List of Down regulated miRNAs					
miR-27b ↓	Causes proliferation, migration, and invasion. Induces tumor growth in vivo. Activates the EMT process and metastatic pathways	*ITGA5*	TSCC cell lines, saliva, and xenograft tumor model	Therapeutic target	[67]
miR-204 ↓	Regulates cancer stemness, cell proliferation, and metastasis	*CXCR4*, *CDC42*, *RAB22A*, *EZR*, *Slug and Sox4*	OSCC cell lines, patient saliva, plasma samples, and nude mice model	Biomarker	[30,68,69]
miR-100 ↓	Plays role in the development or progression of the disease, and may contribute to the loss of sensitivity to ionizing radiation	*MMP13*, *ID1*, *FGFR3*, *EGR2*	OC cell lines	Therapeutic target	[70]
miR-29a ↓	Promoted OSCC cell invasion and induced drug-resistance in vitro	*MMP2*	OC cell lines and patient tissue and saliva samples	Therapeutic target	[71]
Let-7d ↓	Induces chemoresistance to cisplatin and 5-FU	*Snail and Twist*	OC cell lines and saliva samples	Therapeutic target	[27]
miR-203 ↓	Causes tumor proliferation and shows resistance to drugs. Also regulates apoptotic signaling	*BMI-1*, *PI3KCA*, *YES-1*, *SEMA6A*,	Plasma samples and cell lines	Biomarker (diagnosis and prognosis), anti-cancer therapeutics	[72,73,74,75]
miR-200 ↓	Regulate EMT leading to OSCC progression	*ZEB1*, *ZEB2*	Clinical specimens	-	[76]
miR-133a ↓	Regulates tumor proliferation and differentiation, as well as showing antiapoptotic characteristics	*PKM2*, *GSTP1*	Patient samples and cell lines	Therapeutic target	[77,78]
miR-133b ↓	Regulates the proliferation rate of cancer cells as well as the number of apoptotic cells	*PKM2*	Patient samples and cell lines	Therapeutic target	[77]
miR-138 ↓	Causes cell proliferation and inhibits apoptosis	*GNAI2*	TSCC cell lines and plasma	-	[79]
miR-125b ↓	Regulates tumor proliferation and radioresistance mechanisms	*ICAM2 signaling*	Cell lines and tissue specimens	Prognostic markers	[80]
miR-9 ↓	Cause proliferation of OSCC cells	*CXCR4-Wnt/β-catenin*	Patient plasma sample and cell lines	Therapeutic target	[81]
miR-26a/b ↓	Promotes invasion and migration	*TMEM184B*	OC clinical specimens and cell lines	Therapeutic target	[82]
miR-491-5p ↓	Responsible for poor overall survival of patients, invasion, and metastasis	*GIT1*	OC tissues, plasma, cell lines, and mice model	Biomarker (prognosis)	[83]
miR-375 ↓	Promotes tumor proliferation, migration, and invasion	*CIP2A*, *SLC7A11*, *PDGF-A*	Patient tissue and saliva samples and cell lines	Biomarker (diagnosis), therapeutic target	[84,85,86]
miR-320 ↓	Regulates tumor angiogenesis	*HIF-1α-NRP1-VEGF*	Tissue, saliva, plasma samples, cell lines, and mice model	Therapeutic target	[87]
miR-218 ↓	Contributes to oral carcinogenesis.	*mTOR-Rictor-Akt*	OC cell lines	Therapeutic target	[88]
miR-205 ↓	Regulates cancer development by promoting migration, invasion, and inhibiting apoptosis	*IL-24*, *caspase-3/-7*, *Axin-2*, *TIMP-2*	OC cell lines	Therapeutic target	[89,90]
miR-181a ↓	Cause tumorigenesis via stimulating EMT, and enhances metastatic potential	*K-ras*, *Twist1*	Cell lines	Therapeutic target	[91,92]
miR-145 ↓	Causes cell proliferation and colony formation	*c-Myc*, *Cdk6*	Cell lines and tissue samples	Potential biomarker for diagnosis and therapeutic target	[93]
miR-140-5p ↓	Promotes invasion and migration	*ADAM10*, *ERBB4*, *PAX6*, *and LAMC1*	TSCC cell lines	--	[94]
miR-124 ↓	Causes OSCC progression	*ITGB1*	Cell lines	--	[28]
miR-99a ↓	Causes migration, invasion, and lung colonization in OSCC cells	*IGF1R*	Tissue samples	Therapeutic target	[95]
miR-34a ↓	Promotes tumor growth and angiogenesis	*E2F3*	Cell lines, plasma, and SCID mouse xenograft model	Therapeutic target	[96]
miR-17/20a ↓	Causes tumor progression and migration. Expression is negatively correlated with TNM staging and lymphatic metastasis	*ITGβ8*	Cell lines and tissue and saliva samples	Prognostic marker	[97]
miR-137 ↓	Induces tumor differentiation	*--*	OC tissues, plasma and cells	Potential marker for early diagnosis	[98]
miR-5580-3p ↓	Possibly associated with cell viability, proliferation, and migration	*LAMC2*	Tissue and cell lines	Therapeutic target and potential biomarker	[99]
miR-98 ↓	Causes cell growth and metastasis	*IGF1R*	OC tissues and cell lines	Potential therapeutic target	[100]
miR-1 ↓	Promotes migration and invasiveness in OSCC cells.	*Slug*	OC cell lines and tumor tissues	Therapeutic target	[101]
miR-377 ↓	Promotes OSCC growth and migration	*HDAC9*	Tissue samples and cell lines	Therapeutic target	[102]
miR-23a-3p ↓	It is correlated with more advanced cancerous stage and poorer differentiation of OSCC cell	*FGF2*	OC cell lines and tissue	Prognostic biomarker and therapeutic target	[103]
miR-22 ↓	Increased cell viability, migration, and invasion	*NLRP3*	OC tissues and plasma, cell lines, and nude mice xenograft models	Prognostic biomarker and therapeutic target	[104]
miR-139-5p ↓	Causes tumorigenesis and progression	*HOXA9*	Patients tissue and saliva samples, and cell lines	Therapeutic strategy for the treatment	[105]
miRNA-504 ↓	Facilitates proliferation, causes migration and invasion	*CDK6*	OC animal model and cell lines	Therapeutic target	[106]
miR-106a ↓	Induces proliferation and EMT	*LIMK1*	OC tissues and cell lines	Prognostic factor	[107]
miR-16 ↓	Promotes proliferation and inhibited apoptosis	*AKT3*, *BCL2L2*	OC patients and cancer cell lines	Therapeutic target	[108]
miR-495 ↓	Induces cell proliferation and invasion	*Notch1*	OC tissues and plasma and OC cells	Therapeutic target	[109]
miRNA-329 ↓, miRNA-410 ↓	Promotes proliferation and invasion	*Wnt-7b*	OC cell lines and tissues	--	[110]
miR-132 ↓	Promotes proliferation, invasion, and migration, and confers OSCC cell resistance to CDDP-induced apoptosis in vitro	*TGF-β1/Smad2/3*	Tissues and cell lines	Therapeutic target	[111]
miR-376c-3p ↓	Associated with tumor progression, poorer differentiation, lymphoid metastasis, and lymphovascular invasion.	*HOXB7*	Tissue specimens and OC cell lines	Biomarker (early diagnosis)	[112]
miR-769-5p ↓	Causes cancer progression	*JAK1/STAT3 pathway*	OC tissues and cells	Therapeutic target	[113]
miR-486-3p ↓	Promotes proliferation and inhibits apoptosis	*DDR1*	Tissue, saliva, and plasma samples	Therapeutic target	[114]

Up arrows ↑ indicate upregulation and down arrows ↓ indicate downregulation.

**Table 3:** List of circRNAs involved in oral cancer.

circRNAs	Function on Up- or Down-Regulation	Targets/Associated Pathway	Study Model	Biomarker/Therapy	References
List of up-regulated circRNAs					
circ_0002185 ↑	Promotes the proliferation, invasion, migration, EMT in vitro, and tumor growth in vivo	*circUHRF1/miR-526-5p/c-Myc/TGFB1/ESRP1 feedback loop*	OC tissues and cells	Therapeutic target	[237]
circPVT1 ↑	Induces proliferation by serving as a miRNA sponge	*circPVT1/miR-125b axis*	Tissue samples and cell lines	Biomarker and therapeutic target	[231]
circ_100290 ↑	Promotes glycolysis and cell proliferation in OSCC	*circ_100290/miR-378a/GLUT1*	OC cell lines and tissues	--	[230]
circ_0001742 ↑	Encourages proliferation, migration, invasion, and EMT, and resists apoptosis in TSCC cells	*circ_0001742/miR-431-5p/ATF3 axis*	TSCC tissues and cells	Therapeutic target	[238]
circ_0001971 ↑	Regulates cell proliferation, migration, invasion, apoptosis, and chemosensitivity of OSCC	*circ_0001971/miR-194/miR-204*	Tissues, saliva and cell lines	--	[239]
circDOCK1 ↑	Suppresses cell apoptosis	*circDOCK1/miR-196-5p/BIRC3*	OC cell lines, tissue and saliva samples	Biomarker and therapeutic target	[240]
circFLNA ↑	Contributes to laryngeal squamous cell carcinoma (LSCC) migration	*circFLNA/miR-486-3p*	LSCC tissues and cell lines	Therapeutic target	[241]
circGOLPH3 ↑	Promotes the growth of OSCC in vitro and in vivo as well as up-regulating its cell migration and invasion	*circGOLPH3/miR-1299/LIF axis*	OC cell lines and plasma	Therapeutic target	[242]
circCLK3 ↑	Promotes cell proliferation, migration, invasion, and cell cycle in TSCC cells	*miR-455-5p/PARVA axis*	TSCC tissues and cell lines	--	[243]
circCDR1 ↑	Enhances OSCC cell viability, endoplasmic reticulum (ER) stress, and inhibits cell apoptosis under a hypoxic microenvironment	*AKT/ERK½/mTORsignaling pathway*	OSCC cell lines and mice model	Treatment strategy	[232]
circ_0014359 ↑	Promotes cancer progression	*circ_0014359/miR-149 pathway*	Tissues, cell lines, and xenograft mouse model	Diagnosis and treatment	[244]
circ_LPAR3 ↑	Causes tumor growth and angiogenesis	*Circ_LPAR3/miR-513b-5p/VEGFC/AKT1*	OC cell lines and mice model	--	[245]
circ_SEPT9 ↑	Induced OSCC proliferation, migration, and invasion	*circ_SEPT9/miR-1225/PKN2*	OC cell lines, tissues, and xenograft model	Therapeutic target	[246]
circ_0000199 ↑	Associated with tumor size, lymphatic metastasis, and TNM staging in patients with OSCC	*miR-145-5p and miR-29b-3p* (in silico *study*)	Patient plasma samples and cell lines	Biomarker and potential therapeutic target	[247]
circ_0001883 ↑	May be responsible for cell migration, invasion, and EMT of LSCC	*miR-125-5p/PI3K/AKT axis*	Patient samples and cell lines	Therapeutic target for LSCC treatment	[248]
circ_0005320 ↑	Promotes tumorigenesis	*circ_0005320-miR-486-3p/miR-637 axis*	OC tissues, cells, and nude mice model	Biomarker and therapeutic target	[249]
circ_0001874 ↑, circ_0001971 ↑	Associated with tumorigenesis (development and progression of OSCC)	*miR-661*, *miR-662*, *miR-593-5p*, *miR-107*, *and miR-103a-3p* (targets of circ_0001874), *miR-152-5p*, *miR-103a-3p*, *miR-107*, *miR-505-3p*, *and miR-9-5p* (targets of circ_0001971)	Patient sample (saliva)	Diagnostic, prognostic, biomarker and therapeutic target	[250]
circ_0011946 ↑	Causes proliferation, migration, and invasion, as well as restricted apoptosis	*miR-216a-5p/BCL2L2 axis*	OC tissues and cell lines	--	[251]
circ_0001461 ↑	Promotes cell proliferation, migration, and invasion. Promotes resistance to TNF-α-induced apoptosis	*miR-145/TLR4/NF-κB axis*	OC cells and tissues	--	[252]
Circ_DHTKD1 ↑	Promotes tumor growth and metastasis of OSCC	*miR-326/GAB1 axis*	OC cell lines, tissues, and xenograft model	Therapeutic target for clinical diagnosis	[253]
Circ_IGHG ↑	Induces EMT and promotes cancer progression	*miR-142-5p/IGF2BP3 Signaling*	OC cell lines	Diagnosis and treatment	[254]
circ_002178 ↑	Promotes the proliferation and migration	*Akt/mTOR pathway*	OC tissues and cell lines	--	[255]
Circ_VAPA ↑	Promotes the proliferation, migration, and invasion	*miR-132/HOXA7 axis*	OC tissues and cells	Prognosis	[256]
circ-LRP6 ↑	Mediates EMT and autophagy	*-*	OC cells and tissues	--	[257]
List of down regulated circRNAs					
circ_0000140 ↓	Correlated negatively with poor prognostic outcomes in OSCC patients	*miR-31/LATS2 axis of Hippo signaling pathway*	Tissue, cell lines, and xenograft mouse model	Therapeutic target	[258]
circ-PKD2 ↓	Reduced expression in OSCC patients is significantly correlated with aggressive tumor characteristics	*circPDK2/miR-204-3p/APC2*	OC tissue samples and cell lines	Therapeutic target	[236]
circ_0005379 ↓	Its expression is inversely correlated with tumor size and differentiation	*EGFR pathway*	OC patient samples, cell line, and xenograft mouse models	Therapeutic target	[234]
circ_0004491 ↓	Reduced expression is associated with lymph node metastasis, and may facilitate OSCC cell invasion and migration	*circ_0004491/miR-155-5p/SIRT1*	OC tissues and cells	Potential biomarker	[259]
circSPATA6 ↓	Lower expression in OSCC cells fails to impede migration and invasion and facilitate cell cycle arrest and apoptosis	*miR-182/TRAF6 axis*	Cell lines and xenograft tumor model	Targeted therapy	[260]
circ_0086414 ↓	Its lower expression promotes the processes of growth and metastasis in OSCC	*AMPK and cAMPsignaling pathway*	OC tissues and cells	Diagnostic biomarker and OSCC therapy	[261]
circ_0008309 ↓	Associated with pathological differentiation of OSCC patients	*hsa_circ_0008309-miR-136-5P/hsa-miR-382-5P-ATXN1*	OC cell lines	Therapeutic biomarker	[262]
circGDI2 ↓	Fails to serve as a repressor to restrain OSCC malignancy and, thus, regulate OSCC progression	*miR-454-3p/FOXF2 Axis*	OC tissues, cells, and mice model	Biomarker for targeted OSCC therapy	[263]
circ-KIAA0907 ↓	Fails to inhibit migration, invasion, glycolysis, and promote apoptosis, thereby leading to OSCC progression	*miR-96-5p/UNC13C axis*	OC tissues	Potential target for OSCC treatment	[264]
circ_0007059 ↓	Determined to alter cell growth	*AKT/mTORsignaling*	SCC15 and CAL27 cells	Prognostic/therapeutic target	[233]
circ_0004872 ↓	Reverse the promoting effect of miR-424-5p overexpression on the process of OSCC cells	*sponges miR-424-5p*	OC cell lines	Early diagnosis and targeted therapy of OSCC	[265]
circ_0092125 ↓	Associated with clinicopathological factors in OSCC patients, including tumor size, TNM stage, and lymph node metastasis	--	OC cells and tissues	Biomarker of the OSCC prognosis	[266]
circRNA-102450 ↓	Low expression is associated with the tumor metastatic properties and act as a tumor suppressor	*circRNA-102450/miR-1178 axis*	Patient plasma samples	Biomarker	[267]
circ_0072387 ↓	Associated with cell proliferation, migration, invasion, EMT, and glycolysis in OSCC	*miR-503-5p*	Patient sample, OSCC cells, and tissues	Therapeutic target	[268]

Up arrows ↑ indicate upregulation and down arrows ↓ indicate downregulation.

**Table 4:** List of snoRNAs involved in oral cancer.

snoRNAs	Function	Targets	Study Model	Biomarker/Therapy	References
SNHG1 ↑	Promotes TGFβ1-Induced EMT, migration, and invasion of TSCCs	*SNHG1/miR-194-5p/MTFR1 Axis*	TSCC cell lines	Therapeutic target	[275]
SNHG3 ↑	Facilitates cell proliferation and migration in OSCC, and regulates HOXB8	*transcription factor Y subunit gamma*, *SNHG3/miR-2682-5p axis*	OC cell lines	Biomarker	[272,273]
SNHG15 ↑	Increases growth, and facilitates malignant behaviors, of OSCC cells	*miR-188-5p/DAAM1*	OC cell lines	Treatment purpose	[274]
SNHG16 ↑	Enhances progression and carcinogenesis	*-*	CAL-27 and TSCCA cells	--	[276]
SNHG17 ↑	Accelerates proliferation and metastasis of OSCC cells, while reducing apoptosis	*miR-375/PAX6 axis*	CAL-27 and Tca8113 cells	--	[277]
SNHG20 ↑	Enhances the progression of OSCC	*miR-29a/DIXDC1/Wnt regulatory axis*	SCC9 and SCC15 cells	A theoretical basis for the treatment	[162]

Up arrows ↑ indicate upregulation and down arrows ↓ indicate downregulation.

**Table 5:** ncRNAs derived from exosomes, blood, serum, and saliva.

ncRNA Name	Sample	Expression Level in OC vs. Normal Cells	References
miR-8485	Chondrocyte-derived exosomes	Higher	[304]
miRNA-1307-5p	Salivary exosomes	Higher	[301]
miR-200c-3p	Serum exosomes	Higher	[305,306]
miR-143 and miR-221	Plasma exosomes	Lower and higher, respectively	[307,308]
miR-21	Plasma/seminal exosomes	Higher	[309]
miR-31-5p	Macrophage-derived exosomes	Higher	[310]
miR-24-3p	Salivary exosomes	Higher	[311]
miR-382-5p	Fibroblast-associated exosomes	Higher	[66]
miR-10b	Plasma	Higher	[312]
miR-29a-3p	Serum	Higher	[313]
miR-486-5p	Plasma, salivary exosomes	Higher (in Stage II)	[314]
miR-155	Exosomes	Higher	[315]
miR-142-3p	Exosomes	Higher	[316]
miR 455-5p and miR153	Blood plasma	Higher and lower, respectively	[317]
miR-200b-3p, miR-483-5p, miR-425-5p, miR-374b-5p, miR-191-5p, miR-let-7c, miR-29a, miR-103, miR-1234, miR-638, miR-572, miR-22, miR-29b, miR-24-3p, miR-223, miR-20a, miR-29c, miR-17, miR-196a	Blood	Higher	[300]
miR-187, miR-9, miR-223, and miR-29c		Lower	
miR-494	Whole blood	Higher	[318]
miR-16 and let-7b	Serum	Higher	[319]
miR-34a-5p	Cancer-associated fibroblast-derived exosomes	Lower	[320]
miR-3651	Whole blood	Lower	[321]
lncRNAs MAGI2-AS3 and CCDC144NL-AS1	Serum exosomes	Higher	[303]
lncRNA TIRY	Cancer-associated fibroblast-derived exosomes	Higher	[302]
lncRNA ADAMTS9-AS2	Saliva, exosomes	Lower	[322]
circ_0069313	Exosomes	Higher	[323]
circ_0000199	Serum exosomes	Higher	[247]
circ_0026611	Serum exosomes	Higher	[324]
circ_0001874	Salivary exosomes	Higher	[250]
circ_0001971	Salivary exosomes	Higher	[250]

## Abbreviations

OC: Oral Cancer, OSCC: oral squamous cell carcinoma, HNSCC: head and neck squamous cell carcinomas, TSCC: tongue squamous cell carcinoma, LSCC: laryngeal squamous cell cancer, SACC: salivary adenoid cystic carcinoma, ncRNA: non-coding RNA, miRNA: microRNA, lncRNA: long non-coding RNA, CircRNA: circular RNA, piRNA: PIWI-interacting RNA, snoRNA: small nucleolar RNA, siRNA: small interacting RNA, TSG: tumor suppressive gene, mRNA: messenger RNA, Pri-miRNA: primary microRNA, Pre-miRNA: precursor microRNA, EMT: epithelial–mesenchymal transition, snoRNP: small nucleolar protein, MALAT1: metastasis-associated lung adenocarcinoma transcript 1, NEAT1: nuclear paraspeckle assembly transcript 1, CCAT1: colon cancer associated transcript 1, EIciRNA: exon–intron circRNA, tricRNAs: tRNAintronic circular RNA, ecircRNAs: exonic circular RNAs, RBP: RNA-binding protein, LK: leukoplakia, OLP: oral lichen planus, OSF: oral submucous fibrosis.
